# Characteristics of an R-Phycoerythrin with Two γ Subunits Prepared from Red Macroalga *Polysiphonia urceolata*

**DOI:** 10.1371/journal.pone.0120333

**Published:** 2015-03-17

**Authors:** Lu Wang, Shumei Wang, Xuejun Fu, Li Sun

**Affiliations:** 1 College of Life Sciences, Yantai University, Yantai, Shandong, P. R. China; 2 College of Photo-electronic Information Science and Technology, Yantai University, Yantai, P. R. China; Duke University Medical Center, UNITED STATES

## Abstract

An R-phycoerythrin (R-PE) was isolated by gel filtrations on Sepharose CL-4B and Sephadex G-150 from the phycobiliprotein extract of the marine red macroalga *Polysiphonia urceolata* Grev and further purified by ion exchange chromatography on DEAE-Sepharose Fast Flow. The purified R-PE showed three absorption peaks at 498 nm, 538 nm, 566 nm and one fluorescent emission maximum at 577 nm. Although the R-PE showed a single band on the examination by native PAGE, it exhibited two very close bands at pH about 4.7 in native isoelectric focusing (IEF). Polypeptide analysis of the R-PE demonstrated that it contained four chromophore-carrying subunits, α^18.2^, β^20.6^, γ^31.6^ (γ'), γ^34.6^ (γ), and no colorless polypeptide; its subunit composition was 6α^18.2^:6β^20.6^:1 γ^31.6^:2γ^34.6^. The α and β subunits were distributed within a acidic pH range from 5.0 to 6.0 in denaturing IEF and the γ subunits were in a basic pH range from 7.6 to 8.1. These results reveal that the prepared R-PE may exist in two hexamers of γ (αβ)_3_ γ (αβ)_3_γ' and γ (αβ)_3_ γ'(αβ)_3_ γ and that the R-PE participate in the rod domain assembly of *P*. *urceolata* phycobilisomes by stacking each of its trimer (αβ)_3_ face-to-face with the aid of one γ subunit (γ or γ').

## Introduction

Phycobiliproteins are a family of light-harvesting pigment-protein complexes found widely in cyanobacteria, red algae and some cryptomonads [1, 2, 3, 4]. According to their light absorption properties, phycobiliproteins are classified into three main groups: phycoerythrin (PE; λmax = 565–567 nm), phycocyanin (PC; λmax = 615–620 nm) and allophycocyanin (AP; λmax = 650–652 nm) [1–2]. In cyanobacteria and red algae, these phycobiliproteins assemble to form a supermolecular protein complex, named phycobilisome (PBS), by the aid of related linkers [5,6,7]. The sun light harvested by PBSs from surroundings is transferred from the chromophores of PEs to the ones of PCs, to the ones of APs with an overall quantum efficiency closed to 100% [3,8] and finally to photosystem chlorophylls [5–6,9,10]. As major light-harvesting complexes the PBSs make cyanobacteria and red algae able to grow well under the light environments where green algae are hard to live owing to their exclusively using chlorophyll-proteins to harvest light. Among the phycobiliprotein-containing algae, eukaryotic red algae occupy a critical position in the evolution of oxygen-evolving photosynthetic organisms, for they are an inheritor of prokaryotic cyanobacteria and a predecessor of eukaryotic cryptophytes [11,12]. Although PBSs were firstly found from a red microalga *Porphyridium cruentum* and investigated, the detail understanding of the PBS has been accomplished mostly from the researches on cyanobacterium PBSs [6,7,13,14]. Nonetheless, the feature that red algae is able to grow within and under intertidal zone makes it necessary to reinforce the investigations on red alga PBSs even if the characteristics of red alga PBSs are believed to be similar to those of cyanobacterium ones [5].

The property that red algae are able to grow abundant in deep water is dependent primarily on their high content of PEs which have efficient absorption of light from 450 nm to 570 nm. According to light absorption properties, PEs are divided into three main types: B-phycoerythrin (B-PE; λ_max_ = 565 nm, 546 nm and a shoulder at 499 nm), C-phycoerythrin (C-PE; (λ_max_ = 565 nm) and R-phycoerythrin (R-PE; (λ_max_ = 565 nm, 499 nm and a shoulder/peak at 545 nm) [6, 15–16]. B-PE is prepared from unicellular red alga *P*. *cruentum*, R-PE mainly from red macroalgae but the PE of an R-PE type, CU-PE, also observed in some marine cyanobacteria [17], and C-PE from some cyanobacteria. PEs are commonly composed of 6α, 6β and 1γ subunits [18]. The γ subunit is considered to be a connecter by which two (αβ)_3_ trimers (~120–140 kD) are combined to form a hexamer (α^PE^β^PE^)_3_-γ^PE^-(α^PE^β^PE^)_3_ (~240–260 kD) [19–20]. PEs exist usually in hexamer in solution as well as in PBSs and they are stable in diluting solution more than APs and PCs which exist commonly in trimer (αβ)_3_ [6,16, 21]. The PBSs rich in PEs makes red algae play irreplaceable roles in energy and substance cycle of marine ecological system.

For the red algae with a high PE ratio, the coupling of multiple PE hexamers of the same type with each other is critical to the assembly and stability of PBS rod domains. Because PEs take part in PBS assembly and disassociation in hexamer, the hexamer binding of the PEs with high similarity in configuration between one and another is a key factor to the construction of the rod domains. Although the certain investigations on PEs reported that the PEs prepared from some red algae contained two or three γ subunits [13], no one demonstrated the exact γ functions of each kind: a γ subunit acts either as the linker γ^PE^ of PE hexamer formation by connecting two PE trimers, or as the linker γPERPE of PE hexamer combination by coupling two PE hexamers,[(αPEβPE)3−γPE−(αPEβPE)3]−γPERPE−[(αPEβPE)3−γPE−(αPEβPE)3], in rod domain assembly. Therefore, determining the action of individual γ subunits in PBS rod domains composed of multiple PE hexamers has still been one critical point to deepen and promote the investigation on rod domain assembly of red alga PBSs.

For the investigation on PBS assembly, it is essential to isolate and prepare various linker-containing phycobiliprotein complexes originated randomly from PBS dissociation other than component phycobiliproteins. From the linker-containing complexes, some crucial information on PBS construction can be obtained: 1) the relationships between phycobiliproteins and linkers; 2) the proportions of phycobiliprotein subunits to linkers in the complexes; 3) the ratio of linkers to hexameric and trimeric phycobiliproteins. For example, when a PE linker-containing complex is determined to carry two kind γ subunits, it is implied that one γ subunit may function as the connecter of two PE hexamers and the other should act as the connecter of trimers in PE hexamer formation. Therefore, establishing a procedure favorable to preparing linker-containing phycobiliprotein complexes effectively from the constituents of dissociated PBSs is a key step to the investigation on the construction of red algae PBSs.

Glazer reviewed the methods of phycobiliprotein preparation from several different algae by ion exchange chromatography on DEAE-cellulose DE-52 or adsorption chromatography on hydroxyapatite [22]. For the preparation of phycobiliproteins from marine red macroalgae, Liu and colleagues [23] reported the R-PE preparation from *P*. *urceolata*, by using a mode of ion exchange chromatography developed with a pH gradient on DEAE-Sepharose FF. Niu and colleagues [24] reported a process propared *P*. *urceolata* R-PE by chromatography on Q-Sepharose or hydroxyapatite following expanded-bed adsorption chromatography on phenyl-Sepharose Streamline based on the protein adsorption different in ionic or hydrophobic distribution. The hexameric R-PE of (α^PE^β^PE^)_3_-γ^PE^-(α^PE^β^PE^)_3_ with α and β in 18–20 kD and γ ~34 kD prepared by the both labs was consistent with that of the previous structure characterization of the R-PE crystal from *P*. *urceolata* [20].

This paper reports the investigation on the polypeptide composition of an R-PE marine red alga *P*. *urceolata* and focuses on the function of γ subunits in R-PE hexamer formation and multiple R-PE hexamer combination in rod domain construction. The R-PE, which was prepared by a process of gel filtration combined with ion exchange chromatography on a lab-preparative scale from the marine red alga *P*. *urceolata*, is different from that previously reported in subunit composition and contains two γ subunits. Based on the properties of the prepared R-PE hexamer showed in native PAGE and IEF, and the characteristics of its subunit composition and proportions which were demonstrated by SDS-PAGE, denaturing IEF and mass spectroscopy, tow hexameric model of the R-PE were postulated and the action of the two γ subunits in hexamer formation and in the combination between R-PE hexamers in rod domain assembly was discussed.

## Materials and Methods

### Phycobiliprotein Extraction

*P*. *urceolata* used as an organism material in this work for R-PE study is a widespread red macroalga and not a protected organism species. It grows luxuriantly on the local seaside around Yantai city in the area of Northern Yellow Sea of China. The alga specimens were collected at the beach within the district under the jurisdiction of the city government. For the marine algae which are not protected organism species and are used as samples only for teaching and academic or scientific investigations, there are no regulations on the specimen collection limitation of them from the city government and other organizations of biological diversity protection. Therefore, the specimen collection of *P*. *urceolata* used in the research is not required to apply for a specific permission to government departments or related organizations. In addition, there are no other protected organism species included in this investigation.

Fresh alga samples of *P*. *urceolata* were collected in the intertidal zone from February to March; during this period the alga grows abundant although the temperature of seawater was about 5°C to 15°C [19,25]. The alga cells were disrupted by ultrasonication with Ultrasonic Cell Disruptor (Model TY92-II, Ningbuo, China) in 50 mM phosphate buffer (pH 7.0) as 200–300 ml buffer per 100 g alga; the supernatant was collected by centrifugation at 30,000g for 20 min at 4°C.

### Gel filtration

R-PE in the phycobiliprotein extract was first isolated by gel filtration on Sepharose CL-4B (CL-4B). The phycobiliprotein sample with A_566_ ~2.5–3.0 was loaded on the CL-4B column (5.0 × 60 cm) at 0.5 ml/min, and the loading sample was about 10% of the column volume. The gel filtration was developed at 1.0–1.5 ml/min with the 50 mM phosphate buffer containing 50 mM NaCl and 4% (v/v) Triton X-100. The red fraction rich in R-PE was collected and concentrated by salting out with (NH_4_)_2_SO_4_ at saturation of 65% or by ultrafiltration with membrane capsules of 30/50 kD (30K/50K Minimate^TM^ TFF Capsule, Pall Life Sciences, Michigan, USA). The concentrated R-PE solution with A_566_ ~3.0–4.0 was then loaded on the column of Sephadex G-150 (G-150) (3.5 × 65 cm) at 0.25 ml/min. The column was eluted with 50 mM phosphate buffer at 1.0 ml/min and the R-PE was collected. The G-150 chromatography can be easily scaled up by employing a larger column (5.5 × 70 cm).

### Ion exchange chromatography

The R-PE from the gel filtration on G-150 was further purified by ion exchange chromatography on DEAE-Sepharose FF. The collected R-PE was diluted down to 25 mM phosphate buffer, and then the R-PE with about 250 mg protein was loaded on the column (2.6 × 10 cm) at 0.25 ml/min. After the loaded column was fully washed, it was developed at 0.5 ml/min with a linear ionic strength gradient from 0–400 mM NaCl in 500 ml of 25 mM phosphate buffer. Finally, the column was regenerated with 1.5 M NaCl at 0.5 ml/min.

### Gel electrophoresis

The purified R-PE was examined by native PAGE. The native PAGE was composed of a separating gel of 6.5% (w/v) in pH 7.5 Tris-HCl buffer and a stacking gel of 3% (w/v) in pH 5.5 Tris-phosphate acid buffer; and Tris-barbital buffer was used as electrode buffer (pH 7.0) [19,25]. The protein bends were visualized by emission fluorescence under UV light at 365 nm and by staining with Coomassie Blue G-250 (CB G-250).

SDS-PAGE was employed to analyze polypeptide components of the obtained R-PE [19,25]. The SDS-PAGE consisted of a gradient separating gel from 13% to17% in pH 9.2 Tris-HCl and a stacking gel of 5% in pH 6.8 Tris-HCl. Tris-Gly (pH 8.3) of 50 mM was used as electrode buffer. After the electrophoresis, chromophore-carrying subunits were detected under UV light at 365 after the gel was stained with Zn(SO_4_)_2_, and then the band density of polypeptides was analyzed by gel analysis software LabImage after CB G-250 staining.

### Isoelectric focusing

The purified R-PE was also analyzed by IEF in a pH range from 4.0 to 6.5 under native and denaturing conditions. For the native IEF, a polyacrylamide gel of 5.5% (w/v) with 2.0% (v/v) Ampholine (pH 4.0–6.5) was employed, whereas a gel of 7.0% (w/v) with 2.0% (v/v) Ampholine (pH 4.0–6.5) in 8 M urea was used for the denaturing IEF. The two gels were polymerized by the catalysis of 4.0 μg/ml riboflavin under cool-white light for about 20–30 min. The cathode solution was 0.4 M HEPES for the native IEF and 0.5 M ethanolamine for the denaturing IEF, whereas the anode solution was 0.5 M phosphoric acid for both of the IEFs.

The samples used for the denaturing IEF were prepared as following procedures: 1) the samples were denatured in 20% (w/v) trichloroacetic acid, and then the precipitated polypeptides were washed with 4 mM EDTA-Na_2_ containing 10% (v/v) glycerol to remove trichloroacetic acid; 2) the washed polypeptides were suspended in the sample solution composed of 2% (v/v) Ampholine, 0.5% (v/v) NP-40, 120 mM mercaptoethanol and 8 M Urea, and the suspension was centrifuged at 30,000g for 15 min to eliminate insoluble substances.

The IEFs were performed at a constant voltage from 100–300 V for about 3 h. After the native IEF, the R-PE bands were examined in their natural red color and in their yellow fluorescence at 365 nm. For the denaturing IEF, the subunit bands were observed in their orange fluorescence at 365 nm as well as in their natural red color.

### Mass spectrum Measurements

Subunits in gels from SDS-PAGE were digested with Trypsin (10 ng/μl) in 25 mM ammonium carbonate at 37°C overnight, and the peptides from the digested subunits were extracted in turn with 5% (v/v) trifluoroacetic acid and 50% (v/v) acetonitrile in 2.5% (v/v) trifluoroacetic acid. The peptides resuspended in 0.1% (v/v) trifluoroacetic acid was used as samples for mass spectrum measurements, and α-cyano-4-hydroxycinnamic acid (α-CHCA) of 7 mg/ml in 50% (v/v) acetonitrile and 0.1% (v/v) trifluoroacetic acid was employed as the matrix [26–27]. The peptide fragments of the individual subunits were analyzed by MALDI-TOF mass spectrometry (UltrafleXtreme^TM^ MALDI-TOF/TOF, Bruker Daltonics In.). The parameters set for the mass spectrum measurement were [27–28]: laser light: 337 nm; acquisition mode: shots/sub-spectrum 50, total shots/spectrum 1000; resolution: 50,000; mass tolerance: 0.5–0.1 Da; Min S/N: 100; mass range: from 500 Da to 3500 Da.

### Spectrum Measurements

Absorption spectra of the various R-PE samples obtained from a certain corresponding step of the preparation process were recorded with UV-1900 spectrometer (Beijing Purkinje General Instrument Co., Beijing, China) in pH 7.0 phosphate buffer. Fluorescence spectra of them were examined with Cary Eclipse fluorescence spectrometer (Varian Inc., Palo Alto, California, USA) in pH 7.0 phosphate buffer.

## Results

### Isolation of R-PE from *P*. *urceolata*

**Fig 1:**
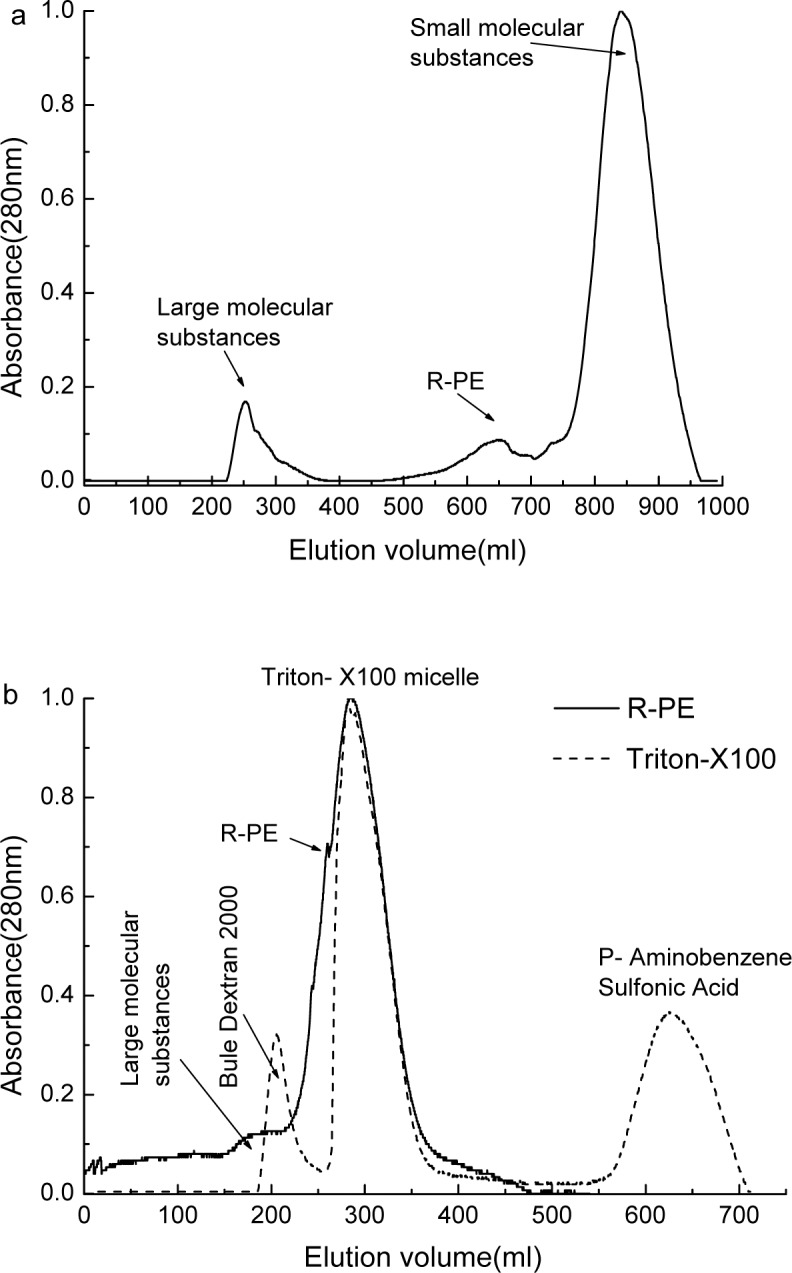
The isolation of the R-PE extracted from marine red alga *P*. *urceolata* by gel filtrations. (a) The gel filtration on Sepharose CL-4B which was developed with 50 mM phosphate buffer (pH 7.0) containing 4% (v/v) Triton X-100; (b) the gel filtration on Sephadex G-150 (solid line) developed with 50 mM phosphate buffer (pH 7.0) and the chromatography of Triton X-100 micelle on Sephadex G-150 (dash line) developed with 50 mM phosphate buffer (pH 7.0).

The chromatogram (Fig. 1a) exhibited that the R-PEs were successfully separated from large molecular substances in dark red color and small molecular substances in light yellow by the gel filtration on Sepharose CL-4B. The dark red substances showed strong adsorption on the CL-4B column even if the column was eluted with 200 mM NaCl in 50 mM phosphate buffer. Nevertheless, this non-specific adsorption was efficiently overcome by adding Triton X-100 in the elution buffer.

By the gel filtration on G-150 (Fig. 1b), the R-PEs in the sample from the CL-4B column were adequately separated from R-PC and AP trimers other than from the remaining large and small molecular substances. There was no observed substance to adsorb on the G-150 column, but a fraction of Triton X-100 micelle was showed to overlap with that of the R-PEs (Fig. 1b). Because of carrying no ionic groups, Triton X-100 micelle hardly bind to ion exchanger media; therefore they are easily isolated from R-PEs by the column washing after sample loading.

**Fig 2:**
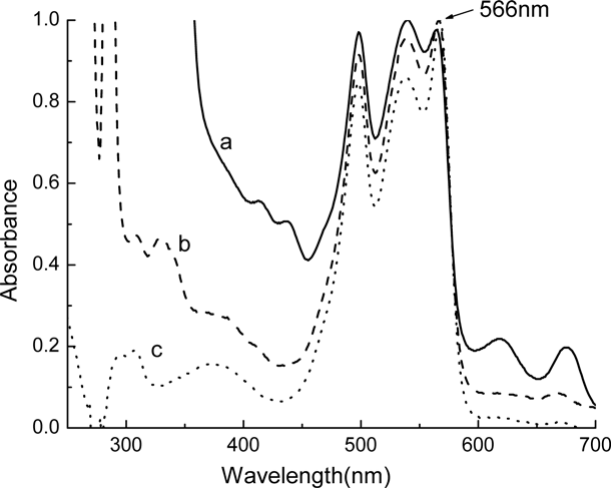
Absorption spectra of three R-PE samples in pH 7.0 phosphate buffer. (a) The phycobiliprotein extract; (b) the R-PE fraction from the gel filtration on Sepharose CL-4B; (c) the R-PE fraction from the gel filtration on Sephadex G-150. The spectra were normalized to the absorbance at 566 nm.

Absorption spectra of the phycobiliprotein extract, the R-PE fractions from the two gel filtrations, as shown in Fig. 2, demonstrated that they effectively removed a large number of substances which have UV-light (≤400 nm) absorption. Furthermore, the R-PEs were also adequately separated from R-PCs, APs and some chlorophyll-proteins which have light absorption in range from 600 nm to 700 nm (Fig. 2). After these isolations, the A_566_/A_280_ ratio of the R-PE samples increased from 0.4 to 0.9.

### Purification of the R-phycoerythrin

**Fig 3:**
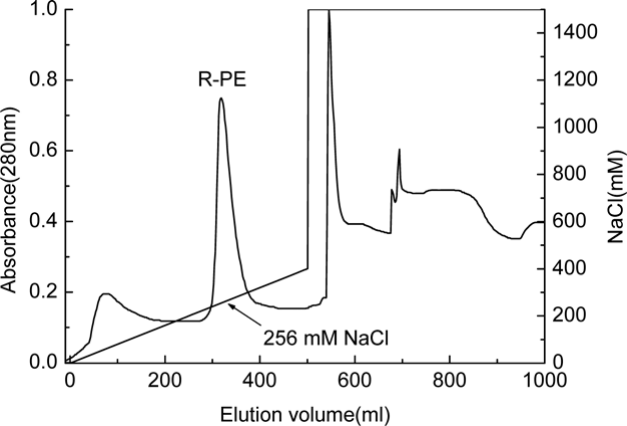
The purification of the isolated R-PE by the ion exchange chromatography on DEAE Sepharose FF. The chromatography was developed at 0.5 ml/min with a linear gradient of NaCl from 0–400 mM in 500 ml of 25 mM phosphate buffer (pH 7.0). After the gradient elution, the column was eluted with 1500 mM NaCl in 25 mM phosphate buffer at 0.5 ml/min.

The R-PE from the gel filtration was finally purified by the ion exchange chromatography on DEAE-Sepharose FF. As shown in Fig. 3, a large R-PE fraction occurred at the elution volume about 319 ml which corresponded to NaCl concentration about 256 mM.

**Fig 4:**
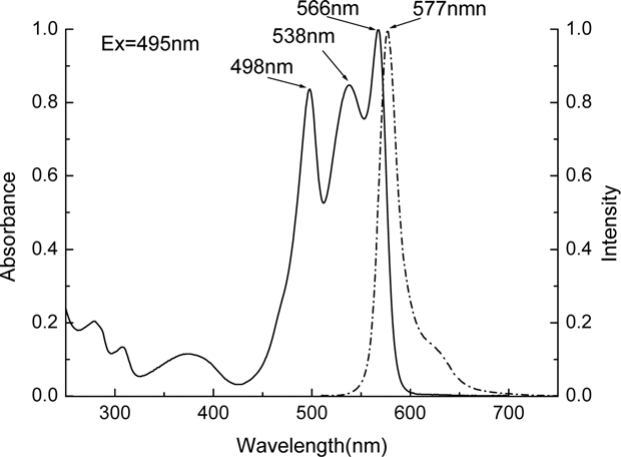
Absorption (solid line) and fluorescent emission (dash line) spectra of the purified R-PE. The ratio of A_566_/A_280_ reaches 5.26. The fluorescent emission spectrum was recorded in pH 7.0 phosphate buffer on the excitation at 495 nm. The spectra were normalized to the absorbance at 566 nm and to the fluorescent emission at 577 nm, respectively.

Fig. 4 shows spectral properties of the purified R-PE. The R-PE has absorption peaks at 498 nm, 538 nm and 566 nm respectively, and shows a fluorescent emission maximum at 577 nm when it is excited at 495 nm. For the purified R-PE, the ratio of A_566_/A_280_ is up to 5.26 which is 0.76 units higher than 4.5. The value 4.5 is the criterion employed commonly for evaluating whether prepared R-PEs have sufficient purity to be used as biological reagents or not [31–32].

**Fig 5:**
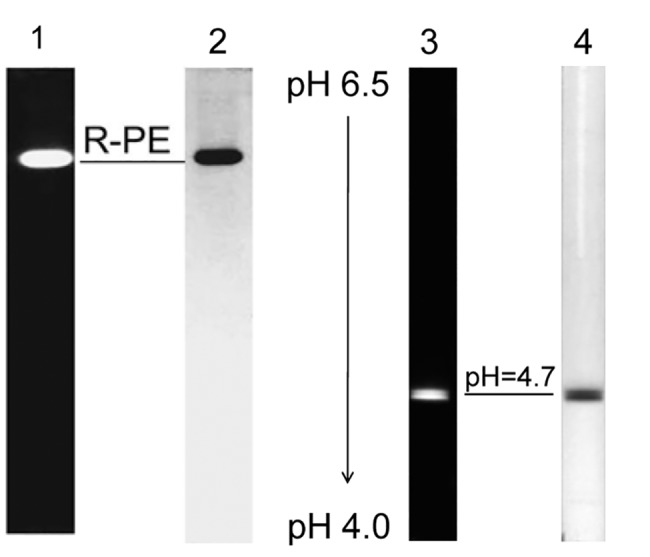
The PAGE (lane 1 and 2) and IEF (lane 3 and 4) of the purified R-PE in native situation. The PAGE had a 6.5% (w/v) separation gel in the neutral buffer system and the IEF had 5.5% (w/v) polyacrylamide gel in a pH range from 4.0 to 6.5. Lane 1 and 3 yellow fluorescent bands of the R-PE in native red color under UV-light at 365 nm; lane 2 and 4 blue bands of the R-PE showed after the gel was stained by Coomassie Blue G-250.

Examination of the purified R-PE by native PAGE and native IEF is shown in Fig. 5. The R-PE exhibited one single band both in yellow fluorescence under UV-light at 365 nm (Fig. 5 lane 1) and in blue color after the gel was stained with CB G-250 (Fig. 5 lane 2). This result demonstrated that the prepared R-PE had high purity. However, in the native IEF (Fig. 5 lane 3 and 4), the R-PE showed two protein bands with very closed pI at about 4.7 in native red color (Fig. 5 lane 3) and in yellow fluorescence under 365 nm and after CB G-250 staining (Fig. 5 lane 4). This fact demonstrates that the prepared R-PE may exist in two hexameric forms. The two hexamers are consistent in subunit composition but different very slightly in pI although they exhibited a homogeneous molecular size in the gel filtrations and a homogeneous ratio of electric charge to molecular mass in the native PAGE.

The same R-PE, in addition, could also be prepared by the same preparation procedures from the PBSs dissociated in 50 mM phosphate buffer. The PBSs from *P*. *urceolata* were prepared by using the sucrose gradient ultracentrifugation used in the previous work of author’s lab [19,33]. But the amount of the prepared R-PE was rather restricted by the limitation of the PBS preparation by ultracentrifugation.

### Polypeptide composition of the purified R-phycoerythrin

**Fig 6:**
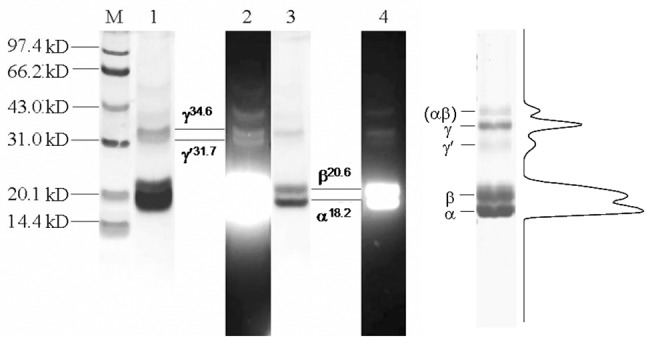
The polypeptide analysis of the purified R-PE by SDS-PAGE. The SDS-PAGE was performed with a gradient separating gel of 13%-17% (w/v) in pH 9.2 Tris-HCl buffer and 4% (w/v) stacking gel in pH 6.8 Tris-HCl buffer. Lane 1 and 3 showed the polypeptide bands after Coomassie Blue G-250 staining. Lane 2 and 4 showed fluorescent bands of the subunits under UV-light at 365 nm after Zn(SO4)2 staining. Lane M showed marker proteins. The right part was the profile curve corresponding to band density and area of the Coomassie Blue G-250 stained pattern of the SDS-PAGE.

As shown in Fig. 6, after the SDS-PAGE electrophoresis, the polypeptides were first visualized under 365 nm after Zn(SO_4_)_2_ staining (Fig. 6 lane 2 and 4) and then observed after CB G-250 staining (Fig. 6 lane 1 and 3). The results showed that the two staining methods gave the same polypeptide band patterns (Fig. 6). This demonstrates that the R-PE is merely composed of chromophore-carrying subunits and contains no colorless liner, for the Zn(SO_4_)_2_ staining specifically visualizes chromophore-containing subunits based on Zn^+2^ enhancing subunit fluorescence emission by coordinating with subunit chromophores. The subunit bands located at 18.2 kD, 20.6 kD, 34.6 kD and 31.6 kD correspond to α, β, γ and γ′ subunits of the R-PE, respectively. Besides the four subunits, other two weak fluorescent bands located at higher molecular mass were proved to be the subunit complexes which are not completely dissociated into subunits.

The right part of Fig. 6 showed the curve profile corresponding to the CB G-250 stained pattern of the SDS-PAGE. On the basis of band density and area, subunit proportions of the purified R-PE are α: β: γ′: γ = 6.05: 6.01: 1.00: 2.07, that is α: β: γ′: γ = 6: 6: 1: 2. These proportions demonstrate that the subunit composition of the R-PE is $\alpha_{6}^{18.\text{2}}\cdot \beta_{6}^{20.\text{6}}\cdot \gamma_{1}^{31.\text{6}}\cdot \gamma_{2}^{34.\text{6}}$. The R-PE molecular mass of 240 kD which was measured by gel filtration on Superdex 200 indicates that it was a hexamer. Accordingly, the prepared R-PE assembles not only in hexameric aggregates, but each hexamer molecule also carries two γ subunits in the proportion of 1: 2. This differs from the R-PE reported by the previous papers [20, 23,34].

**Fig 7:**
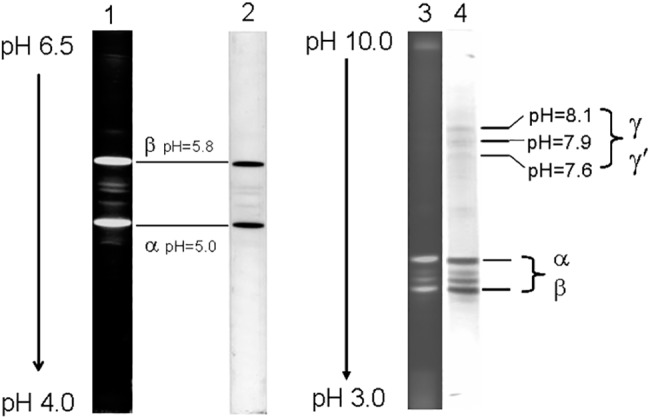
The polypeptide examination of the purified R-PE by IEF in denaturing situation. Lane 1 and 2 in a pH range from 4.0 to 6.5 and lane 3 and 4 in a pH range from 3.0 to 10.0. The polypeptide bands were observed in native red color (lane 2), in blue color after Coomassie Blue G-250 staining (lane 4) and in fluorescence (lane 1 and 3) under UV-light at 365 nm.

**Fig 8:**
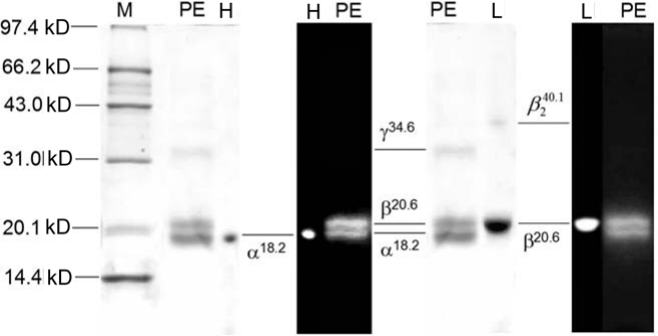
The SDS-PAGE analysis of the subunits occurred at pH 5.0 (low pI band, L) and at pH 5.8 (high pI band, H) in the denaturing IEF. The gel disk of the low pI band (lane L) was cut out from the IEF gel rod and equilibrated in SDS solution at 60C for 30 min, and that of the high pI band (lane H) at 60C for 15 min before they were loaded on the SDS-PAGE with 13% slab gel in pH 9.2 Tris-HCl. The subunits were showed in blue bands after Coomassie Blue G-250 staining and in fluorescence bands under UV-light at 365 nm after Zn(SO_4_)_2_ staining. Lane M showed marker proteins and lane PE was the prepared R-PE.

In the denaturing IEF in a pH range from 4.0 to 6.5 and from 3.0 to 10.0, the R-PE exhibited two thick and three thin colored subunit bands (Fig. 7) and no colorless linker, indicating that the results are identical to those of the SDS-PAGE. As shown in Fig. 8, the thick band situated at pH 5.0 was proved to be β subunit with pI 5.0, whereas the other thick band situated at pH 5.8, subunits, was α subunit with pI 5.8. The 2D-PAGE (not showed) also demonstrated that the thin bands (Fig. 7 lane 3 and 4) between the thick α and β subunits were α or β subunits different in pI. The three bands situated in pH from 7.6 to 8.2 (Fig. 7 lane 3 and 4) were one of the two γ subunits (γ^31.6^ and γ^34.6^).

**Fig 9:**
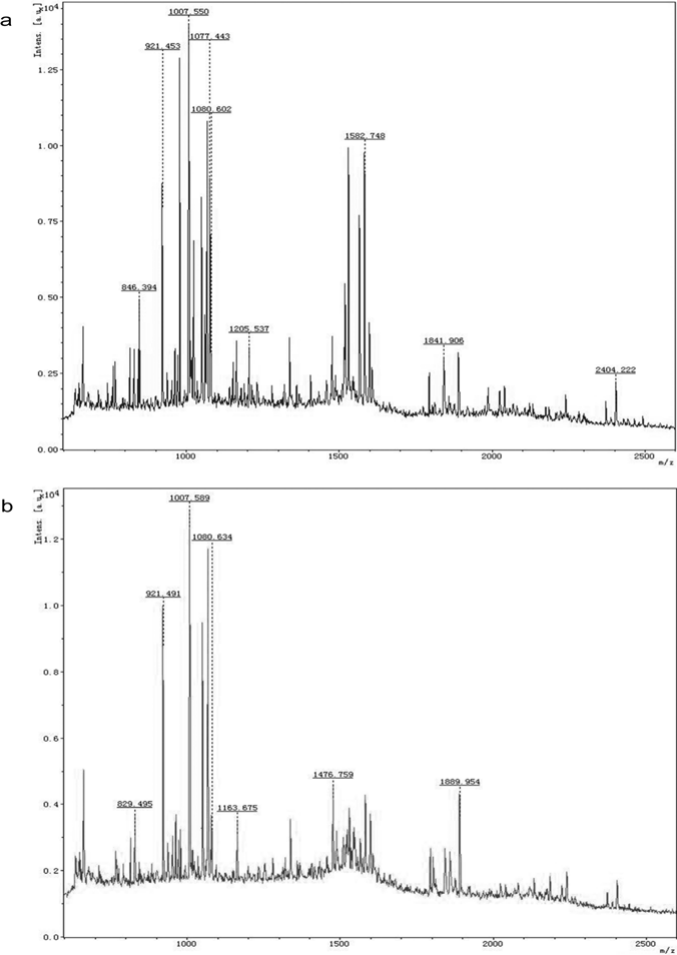
The mass spectra of γ subunit γ^34.6^ (a) and γ^31.6^ (b) measured by MALDI-TOF/TOF mass spectrometry.

Fig. 9 gave the mass spectra of two γ subunit γ^34.6^ and γ^31.6^ obtained by MALDI-TOF/TOF mass spectrometry. The mass spectra adequately exhibited the differences of γ^34.6^ (Fig. 9 a) and γ^31.6^ (Fig. 9 b) in *m/z* peaks. For example, γ^34.6^ showed two *m/z* peaks respectively at 1077.443 and 2404.222 but γ^31.6^ had not; moreover, the peaks of γ^34.6^ and γ^31.6^ occurred respectively at 1205.537 and 1163.675 within 1150–1250, at 1582.478 and 1476.759 within 1400–1600 and at 1841.906 and 1889.954 within 1800–1900. The MS results further demonstrate that γ^34.6^ and γ^31.6^ are two different subunits of the prepared R-PE hexamers.

## Discussion

### The preparation of the R-phycoerythrin from the marine red alga *P*. *urceolata*

On the basis of the present experimental results, the optimized procedures for the preparation of the two γ subunit-containing R-PE from *P*. *urceolata* can be conclusively summarized: 1) the R-PEs salted out from the extracted phycobiliprotein are isolated by the gel filtration on CL-4B developed with 50 mM phosphate buffer containing 0.4% Triton X-100.; 2) the R-PE hexamers from the CL-4B chromatography is separated from R-PC and AP trimers by the gel filtration on G-150 developed with 50 mM phosphate buffer; 3) the R-PEs from the G-150 chromatography are finally purified by the ion exchange chromatography on DEAE Sepharose FF developed with a linear ionic strength gradient of 0.8 mM NaCl per ml. The combination of the two chromatographic modes accords with orthogonality and compatibility requirements of multidimensional chromatography [29–30], and makes the R-PE preparation from *P*. *urceolata* convenience, efficiency and easy scaling-up in practical application. These optimized procedures can also provide a valuable reference for R-PE preparation from other marine red macroalgae.

The chromatographic techniques employed in previous reports on the preparation and structural investigation of the R-PE from *P*. *urceolata* were mainly the chromatography on hydroxyapatite [20], on Q-Sepharose following expanded-bed adsorption chromatography on STREAMLINE Pheny-Sepharose [24] and on DEAE-Sepharose FF developed with a pH gradient from 5.6 to 4.0 [23]. Hydroxyapatite (HAP) chromatography used for protein purification is based on adsorptive interaction between proteins and matrixes, but it is believed that the hydroxyapatite biomolecule interactions are not clearly attributed to any single property of proteins [35]. The complex interactions between HAP and proteins may confer unique separation properties of HAP chromatography, especially when a purification object can not achieve after other modes of chromatography. For hydrophobic interaction chromatography (HIC), the hydrophobic interaction between proteins and the non-polar HIC matrix are promoted by high antichaotropic salt concentration based on the principle of salting out precipitation [30,35–36]. The higher the salt concentration of HIC initial buffers are, the stronger the interaction occurs in principle between HIC matrixes and the hydrophobic patches on proteins [35–37]. In neutral pH environments, Q-Sepharose has electrostatic interaction with loaded proteins more intensive than DEAE-Sepharose FF, for the latter carries smaller surface anions in neutral pH 7.0 with respect to its optimal operation pH range from 6 to 10 [37–38]. Between proteins and matrixes, over strong interactions of any mechanism may impose the irreversible combination of proteins with matrixes, and subsequently decrease the recovery of target proteins. Furthermore, these interactions, especially multiple-site ones, may also bring about some irreversible changes of proteins in conformation, configuration and even polypeptide losing during chromatography processes, especially those processes operated under relatively severe conditions [30,37]. Therefore, the preparation of proteins, particularly multiple polypeptide ones, should be fulfilled under conditions as mild as possible so that the irreversible structural variations, including polypeptide losing, can be diminished to the lowest degree.

For the investigation on polypeptide composition of phycobiliproteins, on phycobiliprotein-linker complexes and on their assembly in phycobilisomes, the key step to the work is the successful preparation of these multiple polypeptide biliproteins or complexes. Thus the biliproteins and complexes can be adequately protected from the interference of their native intactness, including the possible loss of liker polypeptides, during preparation processes. This interference may originate more or less from any chromatographic mode dependent on biomolecule-matrix interactions. Compared with the above mentioned methods [20,23,24] previously used for the R-PE preparation from *P*. *urceolata*, the combination of the gel filtrations on CL-4B and G-150 with the ion exchange chromatography on DEAE-Sepharose FF, which were all performed in neutral pH buffers in the present work, lets the R-PE preparation be carried out under very mild conditions. Therefore, the process is more favorable to preparing the two γ subunit-containing R-PE, a linker-containing complex, from *P*. *urceolata*.

### The subunit composition and assembly of the prepared R-phycoerythrin

The SDS-PAGE (Fig. 6) demonstrated that the prepared R-PE contained two γ subunits, γ^31.6^ and γ^34.6^, as well as α^18.2^ and β^20.6^ and showed no polypeptides without chromophores. The fluorescent bands which exhibited their molecular masses larger than γ^34.6^ were proved to be further dissociated into α^18.2^ and β^20.6^ after they were incubated in SDS solution again. This indicated that they were some remained complexes of α^18.2^ and β^20.6^. The denaturing IEF revealed that all of the α and β subunits had pI in the range from 5.0 to 5.8 (Fig 7 lame 1 and 2), whereas γ^31.6^ and γ^34.6^ subunits showed pI in the range from 7.6 to 8.1 (Fig 7 lame 3 and 4). These facts provide evidence that α^18.2^ and β^20.6^ are acidic polypeptides, but γ^31.6^ and γ^34.6^ which function as linkers in the assembly of R-PE hexamers and PBS rods [16, 19–20,39] are basic ones. The basic feature of the γ subunits is consistent with the common knowledge that linker polypeptides are believed to have basic polypeptide characteristics which were determined based on amino acid sequences of some linker polypeptides [6, 39–40].

Because the difference in pI between α and β is only about 0.8 pH unit, between the α and β subunits the interaction of other types besides electrostatic interaction may inevitably play significant roles in the formation of monomer (αβ) and trimer (αβ)_3_ of the prepared R-PE hexamer. By contrast the electrostatic interaction between acidic α/β and basic γ/γ′ should play an important role in hexamer formation of the prepared R-PE. The stability of hexameric R-PEs more than that of trimeric PCs and APs is attributed to the participation of γ/γ′ subunits in the aggregation of R-PE hexamers by fastening two trimer (αβ)_3_ together in face-to-face.

On the basis of the subunit composition of the prepared R-PE (Fig. 6), $\alpha_{6}^{18.\text{2}}\cdot \beta_{6}^{20.\text{6}}\cdot \gamma_{1}^{31.\text{6}}\cdot \gamma_{2}^{34.\text{6}}$, each hexamer of the R-PE contains one copy of γ^31.6^ and two copies of γ^34.6^. This composition matches with two possible forms of hexamers: 1) $\gamma_{1}^{34.\text{6}}\cdot \alpha_{3}^{18.\text{2}}\beta_{3}^{20.\text{6}}\cdot \gamma_{1}^{34.\text{6}}\cdot \alpha_{3}^{18.\text{2}}\beta_{3}^{20.\text{6}}\cdot \gamma_{1}^{31.\text{6}}$where the two trimers carrying different γ subunits, $\gamma_{1}^{34.\text{6}}\cdot \alpha_{3}^{18.\text{2}}\beta_{3}^{20.\text{6}}$ and $\alpha_{3}^{18.\text{2}}\beta_{3}^{20.\text{6}}\cdot \gamma_{1}^{31.\text{6}}$, are connected by another γ^34.6^; 2) $\gamma_{1}^{34.\text{6}}\cdot \alpha_{3}^{18.\text{2}}\beta_{3}^{20.\text{6}}\cdot \gamma_{1}^{31.\text{6}}\cdot \alpha_{3}^{18.\text{2}}\beta_{3}^{20.\text{6}}\cdot \gamma_{1}^{34.\text{6}}$ where the two trimers of $\gamma_{1}^{34.\text{6}}\cdot \alpha_{3}^{18.\text{2}}\beta_{3}^{20.\text{6}}$ are coupled by γ^31.6^. The two bands with very close pI ~4.7 in the naive IEF (Fig. 5 lane 3 and 4) support the existence of the prepared R-PEs in two hexamer forms. Considering that γ subunit is situated itself in the central cavity of phycobiliprotein trimers and functions as the trimer-trimer linkers in PE hexamer formation [6,16,20,39,41,42,43], the three γ subunits of the prepared R-PE give little contribution to the molecular mass (or size) of hexamers; therefore, the 240 kD molecular mass of the prepared R-PE hexamer determined by gel filtration is dependent more on disk-shape (α_3_β_3_)_2_ and about 100 kD less than the sun of the subunits determined as SDS-polypeptide complexes in rod-shape form by SDS-PAGE.

In conclusion, there are a few striking points which need to be accentuated by contrast to the previous reports [20,23–24,34, 42–43]: 1) the successful preparation of the two γ subunit-containing R-PE from *P*. *urceolata* by the combination of the gel filtrations on CL-4B and G-150 with the ion exchange chromatography on DEAE-Sepharose FF, where all of the chromatographic procedures were performed under mild conditions; 2) the obtained result that the γ subunits of the prepared R-PE all showed their pIs in a pH range from 7.6 to 8.1 is consistent with the commonly accepted fact that linker polypeptides have basic pIs derived from their amino acid compositions; therefore, within the prepared R-PE hexamer the interaction between the α/β subunits with the two different γ ones should be more possibly dependant on electrostatic interaction, but between the α and β subunits different in pI only about 0.8 pH unit hydrophobic interaction may certainly play role in their combination [6–7, 40,44]; 3) the subunit composition of the prepared R-PE hexamer containing one copy γ^31.6^ and two copies γ^34.6^, $\alpha_{6}^{18.\text{2}}\cdot \beta_{6}^{20.\text{6}}\cdot \gamma_{1}^{31.\text{6}}\cdot \gamma_{2}^{34.\text{6}}$, demonstrates that each of trimers is stacked face-to-face with the aid of a γ subunit acting as a rod linker in the rod domain assembly of *P*. *urceolata* PBSs. 4) Considering that the R-PE hexamer $\left(\alpha_{3}^{18.\text{2}}\beta_{3}^{20.\text{6}})\gamma_{1}^{34.\text{6}}(\alpha_{3}^{18.\text{2}}\beta_{3}^{20.\text{6}}\right)$is the more stable hexamer because it has been the hexameric R-PE commonly prepared from *P*. *urceolata* by the previously researches, this fact also reveals that the γ subunit (γ^31.6^) more likely functions as the rod linker between R-PE hexamers in the rod formation, whereas the other γ subunit (γ^34.6^) mostly functions as the linker of trimer-trimer connection in the R-PE hexamer formation. The results reported here have certain advantages of promoting investigation on phycoerythrin aggregation and rod assembly of the phycobilisome from red macroalgae.
